# In vitro antibacterial activity of bioactive glass S53P4 on multiresistant pathogens causing osteomyelitis and prosthetic joint infection

**DOI:** 10.1186/s12879-018-3069-x

**Published:** 2018-04-03

**Authors:** Mateus Trinconi Cunha, Maria Aparecida Murça, Stanley Nigro, Giselle Burlamaqui Klautau, Mauro José Costa Salles

**Affiliations:** 1 Division of Infectious Diseases, Department of Internal Medicine, Santa Casa de São Paulo School of Medical Sciences, São Paulo, Brazil; 2 Department of Laboratory Medicine and Pathology, Santa Casa de São Paulo School of Medical Sciences, São Paulo, Brazil; 3 Hospital da Irmandade da Santa Casa de Misericórdia de São Paulo, Rua Dr Cesário Mota Jr 112, CEP, São Paulo, SP 01303-060 Brazil

**Keywords:** Bioactive glass S53P4, Antibiotic-loaded polymethylmethacrylate, Osteomyelitis, Implant-associated infections, Multidrug resistance, Bacterial infection

## Abstract

**Background:**

Conventional local treatment for medullary osteomyelitis (OM) includes insertion of antibiotic-loaded polymethylmethacrylate (PMMA) cement. Nevertheless, PMMA may delivery irregular concentration of antibiotic to surrounding tissue. We aimed to compare the in vitro antibacterial activity of Bioactive Glass (BAG) S53P4, which is a compound showing local antibacterial activity, to that of antibiotic-loaded PMMA against multidrug resistant bacteria from OM isolates.

**Methods:**

We studied convenience samples of multidrug resistant (MDR) microorganisms obtained from patients presenting OM and prosthetic joint infection (PJI). Mixtures containing tryptic soy broth (TSB) and inert glass beads (2 mm), BAG-S53P4 granules (0.5–0.8 mm and < 45 mm) and Gentamicin or Vancomycin-loaded PMMA beads were inoculated with methicillin-resistant *Staphylococcus aureus* (MRSA) and methicillin-resistant coagulase-negative *Staphylococcus* (MR-CoNS), *Pseudomonas aeruginosa* or *Klebsiella pneumoniae* isolates. Glass beads (2.0 mm) were used as a control. Antibacterial activity was evaluated by means of time-kill curve, through seeding the strains on blood agar plates, and subsequently performing colony counts after 24, 48, 72, 96, 120 and 168 h of incubation. Differences between groups were evaluated by means of two-way analysis of variance (ANOVA) and Bonferroni’s *t* test.

**Results:**

Inhibition of bacterial growth started soon after 48 h of incubation, reached zero CFU/ml between 120 and 168 h of incubation for both antibiotic-loaded PMMA and BAG S53P4 groups, in comparison with inert glass (*p* < 0.05). No difference regarding time-kill curves between antibiotic-loaded PMMA and BAG S53P4 was observed.

**Conclusions:**

BAG S53P4 presented antibacterial properties as much as antibiotic-loaded PMMA for MDR bacteria producing OM and PJI.

**Electronic supplementary material:**

The online version of this article (10.1186/s12879-018-3069-x) contains supplementary material, which is available to authorized users.

## Background

Osteomyelitis (OM) is a heterogeneous disease with several pathophysiological mechanisms, making diagnosis and therapy difficult. Infection can occur through the haematogenous route, direct exposure of the tissue to infective agents or from areas of contiguous infection [1–3]. The microorganisms most commonly involved are the Gram-positive bacteria *Staphylococcus aureus*, coagulase-negative Staphylococci, *Streptococcus sp*.; followed by Gram-negative bacteria such as *Escherichia coli, Pseudomonas aeruginosa* and *Acinetobacter* spp*.*. An increasing number of cases of OM caused by multiresistant microorganisms, both in hospital and community-acquired infections, have recently been reported [4, 5]^,^. This microbiological profile is most characteristic in OM following surgeries for the treatment of fractures and articular degenerative diseases (osteosynthesis and arthroplasties), or secondary to pressure ulcers. In contiguous OM associated with complex exposed fractures, the presence of polymicrobial infection is also common [6]. The expression of antimicrobial resistance by these agents, coupled with biofilm formation, reduces cure rates and increases disease morbidity, presenting a challenge to the current treatment of OM.

To maintain bone tissue free of microorganisms after debridement, a combination of systemic and local antimicrobial therapy - by filling the bone cavity with antibiotic carriers such as polymethyl-methacrylate (PMMA) - is currently used. Although it is the only material currently approved by the Food and Drug Administration (FDA) in the treatment of osteomyelitis, antibiotic-loaded PMMA has some limitations. Because it is not biodegradable, its use requires several surgical procedures, which may result in loss of bone mass [7]. In addition, the surface of the polymer can act as a substrate for the formation of biofilms, leading to a reduction in the effectiveness of the antimicrobials [8]. Previous studies have also shown that the use of PMMA as a vehicle for local antibiotic therapy can produce inconsistent release of the drug, with a consequent reduction in the efficacy of therapy and induction of bacterial resistance [9, 10]^,^. Thus, it is becoming increasingly important to develop alternative treatment strategies and materials for those infections that are difficult to control.

Bioactive Glass (BAG) S53P4 is one of the latest materials to be studied, with its properties presenting solutions to some of the weaknesses of the treatment currently advocated with PMMA or cement. It consists of several ionic compounds (SiO_2_, Na_2_O, CaO, P_2_O_5_), and when implanted in the cavity after debridement, releases alkali ions, causing an abrupt increase in pH and, in the process, becoming hydroxyapatite [11, 12]^,^. This allows both inhibition of bacterial growth through the osmotic and acid-base imbalance generated – not being dependent on antibiotics to occur [13, 14]^,^ −, and the formation of new bone matrix, not requiring further implant removal procedures. It is important to notice that, although BAG remains present considerable periods of time after implantation, theoretically being able to act as foreign material, there are no reported foreign body reactions or infections involving S53P4 to date [15]. We aimed to analyze the in vitro antibacterial activity of BAG compared to antibiotic-loaded PMMA in multidrug-resistant bacteria strains producing osteomyelitis and prosthetic joint infections.

## Methods

### Study design

In the study, we analyzed the effect of BAG S53P4 (BonAlive Biomaterials Ltd., Turku, Finland) and antibiotic-loaded PMMA (DePuy Smart Set Bone Cement, DePuy GMW Endurance, DePuy International Ltd., UK), on eighteen multidrug resistant bacterial strains isolated from bone tissue and sonicate fluid cultures of patients presenting osteomyelitis and orthopedic-implant associated infections. All strains were previously stored at − 70 °C in 80% glycerol at Santa Casa de São Paulo clinical microbiology laboratory. These pure clinical isolates were five methicillin-resistant *Staphylococcus aureus* strains (MRSA), five methicillin-resistant coagulase-negative *Staphylococcus* strains (MR-CoNS), four carbapenemase-producing *Klebsiella pneumoniae* strains (KPC) and four multidrug-resistant *Pseudomonas aeruginosa* strains (MDR-Pa). We used two control strains, both ATCC stock cultures (*Pseudomonas aeruginosa* ATCC 27853 and *Klebsiella pneumoniae* ATCC 700603). The study was approved by the local Institutional Review Board.

Antimicrobial resistance was evaluated by means of Minimal Inhibitory Concentration (MIC) method for Vancomycin and Polymyxin B, and Disc Diffusion Test for other antibiotics. Details of the antimicrobial resistance profiles can be found in Additional file 1: Tables S1, S2, S3 and S4. For both methods, 3 to 4 randomly selected, dot-shaped Colony Forming Units (CFU) of each bacterial clinical isolate were resuspended (TSB broth, BHI broth or saline) to a turbidity of 0.5 on the McFarland scale (1.5 × 10^8^ CFU/mL). After visual adjustment, the *inoculum* were seeded on Mueller Hinton or Mueller Hinton Blood agar, and antibiotic-impregnated discs and the E-test strips (BioMérieux, Marcy-l'Étoile, France) were placed on the seeded agar surface. The plates were incubated aerobically at 35–37 °C in 5 to 7% CO_2_ for 24 h. After incubation, the diameters of inhibition halos were measured and interpreted according to current Clinical Laboratory Standards Institute (CLSI) standards [16]. MIC measures were read from the scale where the inhibition ellipse’s edge intersected the strip.

In addition, 500 μL of the bacterial suspension was inoculated into three different test tubes: (1) BAG tube containing a 5 mL mixture consisting of TSB broth and BAG S53P4 in granules of 0.4–0.8 mm at a concentration of 0.8 mg/ml; (2) PMMA tube containing 5 mL mixture of TSB broth and antibiotic impregnated PMMA in 2-3 mm beads at a concentration of 0.4 mg/ml (vancomycin 50 mg/g was used for Gram-positive cocci, while gentamicin 25 mg/g was used for Gram-negative); and (3) Glass tube containing 5 mL mixture of TSB broth and previously autoclaved inert glass in 2 mm diameter beads (Plena Lab, Sao Paulo, Brazil).

### BAG preparation

BAG was mixed with TSB broth by means of vortexing for 1 to 2 min and the resulting suspension was incubated for 48 h in a heating chamber at 35–37 °C, followed by verification of pH with indicator strips (MColorpHast™, Merck KGaA, Darmstadt, Germany). This incubation period was used in order to keep the method as similar to other studies in literature as possible [13, 14]. Detection of pH above 10 was considered as evidence of the physicochemical reaction of the BAG and was therefore suitable for use in the experiment [14]. During this phase, there was no sample whose pH was below the threshold.

### Definition of the estimated optimum concentration of BAG

Using two bacterial strains of different species each (*Staphylococcus aureus* and *Klebsiella pneumoniae*), we tested three different concentrations of BAG using four groups of tubes containing 5 mL of suspensions in TSB broth: BAG at concentrations of (1) 400, (2) 600 and (3) 800 mg/mL, and (4) inert glass as control. The strains were suspended at 0.5 on the McFarland turbidity scale and thereafter 500 μL of the suspensions were inoculated in the tubes, resulting in an inoculum of 1,5 × 10^5^ CFU/mL. Additionally, the tubes were incubated aerobically at 35–37 °C in 5–7% CO_2_ for 5 days. Blood agar was seeded with 10 μL drawn from each tube immediately and 24, 48, 72 and 120 h after inoculation of the suspensions, and the results were analyzed by counting the CFU on the plates. The study was conducted in duplicate for each bacterial strain. The lowest concentration to yield no bacterial growth during the observation period was considered the optimum concentration of BAG to be used in the main experiment’s BAG suspensions. Only the tubes containing BAG at a concentration of 800 mg/mL presented complete growth inhibition during the 5 days of analysis.

### Statistical analysis

Analysis of the results was made by plotting a bacterial Time-Kill Curve (log of CFU vs. time). Statistical analysis and graphs were made using STATA software version 13.1 (STATA Corp., Texas, USA). The results were evaluated through two-way ANOVA and the Bonferroni t-test. Values of *P* < 0.05 were considered significant.

## Results

### Comparative analysis of antibacterial activity of BAG in relation to antibiotic-impregnated PMMA

**Fig. 1:**
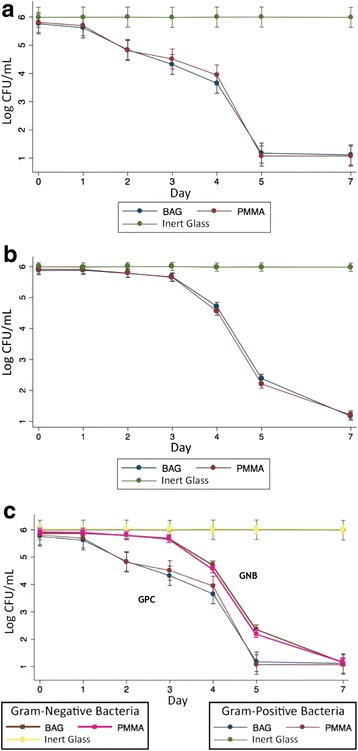
**a** Time-Kill Curve for Gram-Positive Cocci. **b** Time-Kill Curve for Gram-Negative Bacilli. **c** Comparison between Gram-Positive and Gram-Negative Groups’ Time-Kill Curves

**Fig. 2:**
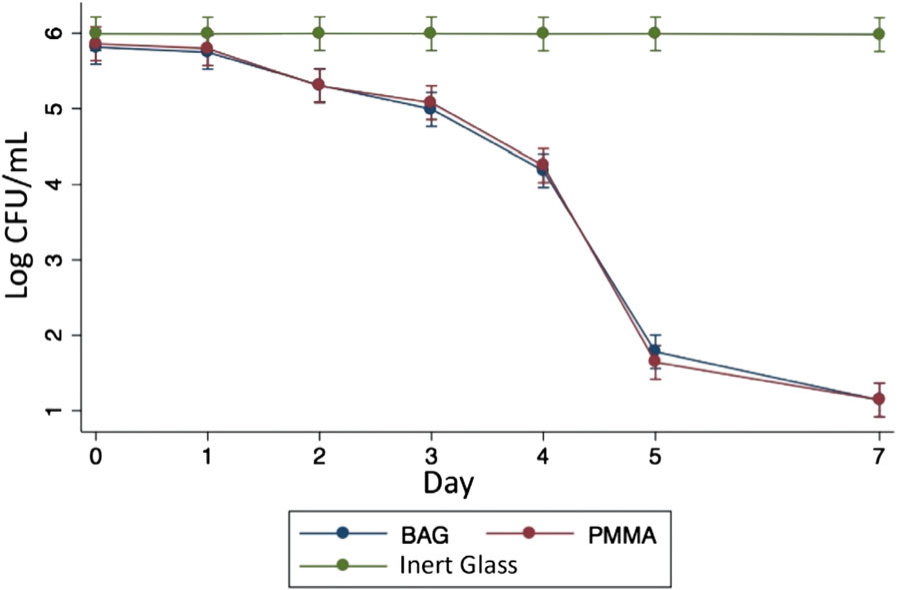
Time-Kill Curve for All Organisms Tested

Bacterial Time-Kill Curves were produced according to the antibiotic used, and Gram staining classification of each bacterium, the total of all groups as well as comparison between them are shown in Figs. 1a-c and 2.

By observing the death curve of the Gram-positive bacteria, BAG’s bacterial growth inhibition is evident, presenting a decrease of approximately one Log in 48 h, 1 additional Log between 48 and 96 h, and approximately three Logs from 96 to 120 h. It can also be seen that in the group under the influence of the BAG there was a statistical difference in comparison with the control group (inert glass beads) (*p* < 0.05), while it remained very similar to the PMMA associated with vancomycin treatment curve, showing no statistically significant difference with the group (Fig. 1a).

In the bacterial death curve of Gram-negative microorganisms, a similar phenomenon can be observed: compared to control, a decrease of approximately one Log was observed in 96 h, 2 additional Logs between 96 and 120 h, and approximately 1 Log from 120 to 168 h (p < 0.05). During the evaluation period, the curves of intervention groups show no statistical difference (Fig. 1b).

The rate of growth inhibition was significantly different between Gram-positive and Gram-negative bacteria (p < 0.05), starting from 48 h of observation until the endpoint of 168 h, in which BAG start killing Gram-positive bacteria faster than Gram-negative bacilli. Although, there were no statistical difference between groups on day 7 of observation. BAG and PMMA presented the same killing curves for both Gram-positive and Gran-negative bacteria. (Fig. 1c).

When combining all bacterial strains, the killing curves showed that both BAG S53P4 and antibiotic-loaded (vancomycin or gentamicin) PMMA showed potent bacterial growth inhibition, starting with approximately 1 Log at 72 h, 1 additional Log between 72 and 96 h, approximately 2 Logs from 96 to 120 h, and 1 more Log between 120 and 168 h. There was no statistical difference between killing curves. (Fig. 2).

## Discussion

The success of the BAG S53P4 intervention in inhibiting bacterial multiplication in this study is in line with the current trend in the literature, which supports the antimicrobial effect of this biomaterial [13–15, 17–26]. In addition, although the literature on the subject is still scarce, our results reinforce the hypothesis of some authors [20] who have concluded that the antibacterial activity of antibiotic-loaded PMMA and BAG S53P4 were equivalent, making this material a viable local alternative treatment for medullary osteomyelitis, with no extra need of antibiotic-based therapy. Moreover, in the clinical setting, BAG has consistently proven to be an effective treatment to patients with OM. In a multicentric study, Lindfors et al. [19] analyzed the use of BAG in the treatment of 116 patients with OM and obtained a 90% success rate, very similar to the 88.9% success rate found by Drago et al. [22] and 91.7% found by Fernando et al. (2017). Furthermore, in the same study, Lindfors et al. found that the use of BAG as a one-stage procedure without local antibiotic therapy as an efficient treatment option when compared to a two-stage treatment using antibiotic-loaded PMMA beads prior to the implantation of BAG.

Although our results have shown efficient microbial inhibitory activity against an inoculum of 1,5 × 10^5^ CFU/mL, comparable to patients with OM that are debrided (models range from 10^4^ to 10^6^ CFU/mL) [27–29], bacterial inhibition did not occur with the same speed as in most studies previously published [13, 14, 17, 20, 25, 26]. In our experiment, PMMA and BAG granules started reducing the bacterial growth only after 24 h, while killing a large number of bacteria took at least 48–72 h after inoculation. In contrast, different studies reported a significant earlier drop (24 to 48 h) in the number of colony forming units when compared the activity of BAG S53P4 with PMMA or calcium sulphate antibiotic beads [17, 18, 20]. This may be explained by the use of test tubes containing the BAG mixtures in this experiment, which reduces the contact surface area between the agent and the culture medium, a factor that may have influenced the release of alkaline ions into the mixture, retarding its effect on bacterial inhibition [30]. In addition to the containers used to hold the mixtures, few other adaptations were necessary to carry out this study. Interestingly, in the present study the bacterial activity of BAG and PMMA demonstrated slightly differences, with a slower bacterial inhibitory activity against Gram-negative bacteria. This phenomenon may be attributed to Gram-negative organisms’ several genetic mechanisms of adaptation to changes in environmental osmolarity and pH [31–33], Furthermore, the pH threshold value of 10 used to indicate BAG suitability is fairly close to *P. aeruginosa* natural habitats’ pH values, that range from 4,5 to 9,5. Nonetheless, after 7 days, both BAG and PMMA were able to eliminate all bacteria.

It is also important to point that, since the colony counts were manually performed, we were unable to determine bacterial CFU values above 3.8 × 10^6^ CFU. This matter is especially relevant to the interpretation of control groups’ time-kill curves in Figs. 1a, b, c and 2, because since the first count (seeded immediately after bacterial inoculation), the plates were almost fully covered in colonies, usually more than 100/cm^2^. In this kind of colony distribution pattern, the maximum CFU count possible is defined by the expression [Petri dish area]/[inoculum concentration]. In our case, 58cm^2^/(1.5 x 10^5^CFU/mL), resulting in about 3.8 × 10^6^ CFU. As a consequence, in Figs. 1, 2, 3 and 4, control groups’ time-kill curve shows a fairly constant bacterial count instead of gradually increasing values. This work, despite its limitations represents one of the first analyses of the antimicrobial activity of BAG S53P4 against clinical species that cause osteomyelitis and prosthetic joint infection from a middle-income country, as well as being the second largest in vitro study in the literature, in which 18 MDR Gram-positive and Gram-negative bacteria were studied. In addition to this, this is the largest in vitro experimental comparison between BAG and the conventional cement-based treatment (PMMA associated with vancomycin or gentamicin) and is of particular importance given its focus on the effect of the intervention on multidrug-resistant species, which have played an increasing role in the etiopathogenesis of osteomyelitis, as can be seen in Table 1.

**Table 1:** Number of individuals of the main studies in the literature and remarks

Authors	Year of Publication	Number of patients	Number of Strains	Remarks
Lindfors N.C. [19]	2017	116	–	C / ^a^
Romanò C.L. [17]	2014	76	–	C / ^a^
Munukka E. [21]	2007	–	29	^a^
Ferrando A. [18]	2017	25	–	C / ^a^
Drago L. [22]	2013	27	20	M / ^a^
Leppäranta O. [23]	2007	–	15	^a^
Lindfors N.C. [24]	2010	11		C / ^a^
Gergely I. [20]	2014	–	4	
Bortolin M. [25]	2015	–	3	B / ^a^
Drago L. [26]	2015	–	3	^a^
Drago L. [14]	2014	–	2	B / ^a^
Coraça-Huber D. [13]	2013	–	1	B / ^a^

*C* study is not in vitro but clinical trial or cohort involving patients, *M* study has both in vitro and in vivo branches, *B* study whose outcome was the analysis of the activity of the BAG against bacterial biofilms; ^a^ study in which there is no comparison with PMMA impregnated with antibiotics

## Conclusions

In conclusion, the results of this study are in line with the trend in the current literature which points to BAG S53P4 being an effective alternative to the current treatment of choice for medullary osteomyelitis. Further prospective studies and clinical trials should be carried out on patients.

## Additional file

Additional file 1: Details of the antimicrobial resistance profiles of all tested microorganisms is described in Tables S1, S2, S3 and S4. **Table S1:** Antibiotic resistant profile of oxacillin-resistant *Staphylococcus aureus* strains. **Table S2**: Antibiotic resistant profile of oxacillin-resistant coagulase-negative *Staphylococcus* strains. **Table S3**: Antibiotic resistant profile of *Pseudomonas aeruginosa* strains. **Table S4**: Antibiotic resistant profile of *Klebsiella pneumoniae strains. (PDF 184 kb)*

## Abbreviations

(KPC): Carbapenemase-Producing *Klebsiella pneumoniae*
ANOVA: analysis of variance
BAG: Bioactive Glass
CFU: Colony Forming Units
CLSI: Clinical Laboratory Standards Institute
FDA: Food and Drug Administration;
MDR: Multidrug Resistant
MDR-Pa: Multidrug-Resistant *Pseudomonas aeruginosa*
MIC: Minimal Inhibitory Concentration
MR-CoNS: methicillin-resistant coagulase-negative *Staphylococcus*
MRSA: methicillin-resistant *Staphylococcus aureus*
OM: Osteomyelitis
PJI: Prosthetic Joint Infection
PMMA: Polymethylmethacrylate
