# Supplementary material for: In vitro antibacterial activity of bioactive glass S53P4 on multiresistant pathogens causing osteomyelitis and prosthetic joint infection
Source: BMC Infect Dis. 2018 Apr 3;18:157. doi: 10.1186/s12879-018-3069-x (PMC5883601; doi:10.1186/s12879-018-3069-x)
Supplement: Supplementary file 1 — Details of the antimicrobial resistance profiles of all tested microorganisms is described in Tables S1, S2, S3 and S4. Table S1: Antibiotic resistant profile of oxacillin-resistant Staphylococcus aureus strains. Table S2: Antibiotic resistant profile of oxacillin-resistant coagulase-negative Staphylococcus strains. Table S3: Antibiotic resistant profile of Pseudomonas aeruginosa strains. Table S4: Antibiotic resistant profile of Klebsiella pneumoniae strains. (PDF 184 kb) [file 12879_2018_3069_MOESM1_ESM.pdf]

Additional tables

Details of the antimicrobial resistance profiles is described in Tables S1, S2, S3 and S4.

Table S1: Antibiotic resistant profile of oxacillin-resistant *Staphylococcus aureus* strains.

	Clinical Case Number				
**Antibiotics**	**SS32**	**SS53**	**SS160**	**SS164**	**SS400**
Penicillin	R[&]	R	R	R	R
Oxacillin	R	R	R	R	R
Teicoplanin	S[a]	S	S	S	S
Vancomycin (MIC[#] µg/L)	0.3	1	0.5	0.5	1
Erythromycin	R	S	S	S	S
Ciprofloxacin	R	R	R	R	R
Clindamycin	R	R	R	R	R
TMP/SMX**	S	S	S	S	S
Tetracycline	S	S	S	S	S

R[&] = Resistant to tested antibiotic; S[a] = Sensitive to tested antibiotic; SMX/TMP** = Sulfamethoxazole / Trimethoprim; MIC[#] = Minimal Inhibitory Concentration value, expressed in µg/L, obtained by means of Etest.

Table S2: Antibiotic resistant profile of oxacillin-resistant coagulase-negative *Staphylococcus strains.*

	Clinical Case Number				
**Antibiotics**	**SS112**	**SS119**	**SS123**	**SS126**	**SS141**
Penicillin	R[&]	R	R	R	R
Oxacillin	R	R	R	R	R
Teicoplanin	S[a]	R	R	R	S
Vancomycin (MIC[#] µg/l)	1	1.5	1	0.5	0.75
Erythromycin	R	R	R	R	R
Ciprofloxacin	R	S	R	R	S
Levofloxacin	R	S	R	R	S
Clindamycin	R	R	R	R	S
SMX/TMP**	R	S	R	R	S
Tetracycline	S	S	S	R	S
Chloramphenicol	R	S	R	R	S

R[&] = Resistant to tested antibiotic; S[a] = Sensitive to tested antibiotic; SMX/TMP** = Sulfamethoxazole / Trimethoprim; MIC[#] = Minimal Inhibitory Concentration value, expressed in µg/L, obtained by means of Etest

Table S3: Antibiotic resistant profile of *Pseudomonas aeruginosa* strains.

	Clinical Case Number/ ATCC strain				
**Antibiotics**	**SS461**	**SS462**	**SS486**	**SS487**	**ATCC 27853**
Gentamicin	R[&]	R	S[a]	R	S
Amikacin	R	R	S	R	S
Ceftazidime	S	S	S	R	S
Cefepime	R	R	S	R	S
Aztreonam	R	R	R	S	S
Meropenem	R	R	R	R	S
Imipenem	R	S	R	R	S
Pip/Taz[b]	R	R	S	S	S
Tic/Clv[c]	R	R	R	S	S
Ciprofloxacin	R	R	S	R	S
Levofloxacin	R	R	S	R	S
Polymyxin (MIC[#] µg/l)	1.0	1.0	0.45	Not Tested*	0.25

R[&] = Resistant to tested antibiotic; S[a] = Sensitive to tested antibiotic; Pip/Taz[b] = Piperacillin / Tazobactam; Tic/Clv[c] = Ticarcillin / Clavulanate**;** MIC[#] = Minimal Inhibitory Concentration, expressed in µg/L, obtained by means of Etest; * = the test was not performed.

Table S4: Antibiotic resistant profile of *Klebsiella pneumoniae strains.*

	Clinical Case Number/ATCC strain				
**Antibiotic**	**SS71 (KPC)**	**SS290 (ESBL)**	**SS313 (KPC)**	**SS443 (ESBL)**	**ATCC 700603**
Ampicillin	R[&]	R	R	R	R
Amx / Clv[d]	R	R	R	S[a]	R
Gentamicin	R	S	R	R	S
Amikacin	S	S	S	R	S
Ceftriaxone	R	R	R	R	R
Ceftazidime	R	R	R	R	R
Cefepime	R	R	R	R	R
Aztreonam	R	R	R	R	R
Meropenem	R	S	R	S	S
Imipenem	R	S	R	S	S
Ertapenem	R	S	R	S	S
Ciprofloxacin	R	R	R	R	R
Levofloxacin	R	R	R	R	R
SMX/TMP**	R	R	R	R	R
Chloramphenicol	R	R	R	R	R
Tigecycline	S	S	R	S	S
Polymyxin (MIC[#] µg/L)	1	1	0.38	1	1

R[&] = Resistant to tested antibiotic; S[a] = Sensitive to tested antibiotic; Amx / Clv[d] = Amoxicillin/Clavulanate; SMX/TMP** = Sulfamethoxazole/Trimethoprim; MIC[#] = Minimal Inhibitory Concentration value, expressed in µg/L, obtained by means of Etest.
